# Complete remission of primary retroperitoneal transitional cell carcinoma after radiotherapy and oral chemotherapy: a case report

**DOI:** 10.1308/003588413X13511609955058

**Published:** 2013-03

**Authors:** K Ichinohe, M Ijima, T Usami, S Baba

**Affiliations:** ^1^Fukuroi Municipal Hospital,Japan; ^2^Kakegawa City Hospital,Japan; ^3^Fukujukai Hiryu Clinic, Hamamatsu,Japan; ^4^Hamamatsu University School of Medicine,Japan

**Keywords:** Retroperitoneal, Carcinoma, Radiotherapy, Chemotherapy, Tegafur, Uracil

## Abstract

Primary retroperitoneal transitional cell carcinomas (TCCs) are extremely rare neoplasms for which prognosis is very poor. We present a case that underwent complete remission after radiotherapy and concurrent oral chemotherapy. A 68-year-old woman presented with acute onset of bloody stool. Urgent colonoscopy only detected haemorrhoids. Subsequent abdominal ultrasonography revealed a mass of 7cm in maximal diameter in the left iliac fossa. Laparotomy disclosed a retroperitoneal mass that could not be dissected and therefore only incision biopsy was performed. After a final diagnosis of primary retroperitoneal TCC, chemotherapy with tegafur-uracil (UFT) was initiated but was not effective. Subsequently, radiotherapy was initiated concurrently with UFT at a total dose of 50Gy in 25 fractions. At 20 months after radiotherapy, the tumour seemed to have completely remitted. At the last follow-up, ten years from radiotherapy, computed tomography revealed no recurrence.

We identified only three single case reports regarding primary retroperitoneal TCC over the last five decades. All patients died from the tumour 8−24 months after diagnosis or treatment. Based on the success of our case, radiotherapy with concurrent oral chemotherapy should be considered as an option for unresected cases.

## Case history

A 68-year-old woman presented at a neighbouring hospital with acute onset of bloody stool in August 2001. She had received a subtotal gastrectomy for a gastric ulcer 20 years previously but was taking no medication at presentation. Physical examination revealed mild tenderness in the left lower abdomen. Urgent colonoscopy disclosed only haemorrhoids. Subsequent abdominal ultrasonography revealed a mass of 7cm in maximal diameter in the left iliac fossa. On pelvic examination, a hard mass was palpated on the left side of the normal-sized uterus and was unmovable. Serum CA125 was increased to 484.2u/ml (normal <35.0u/ml) but CA19-9 and routine blood tests were normal.

**Figure 1:**
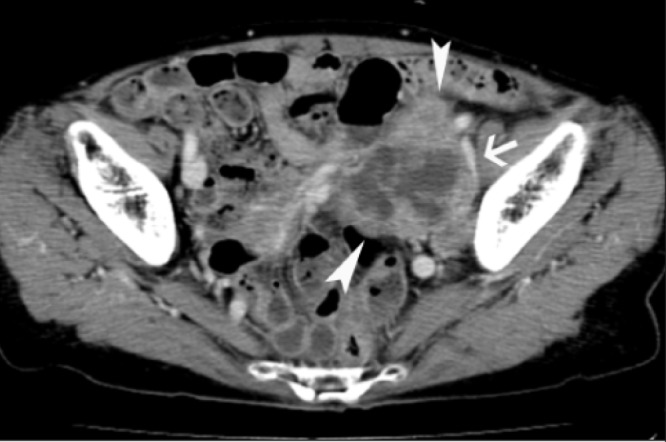
Post-contrast computed tomography of the abdomen at presentation showing a lobulated mass (arrowheads) with cystic components in the left pelvic wall. The mass encases the left iliac vein (arrow).

**Figure 2:**
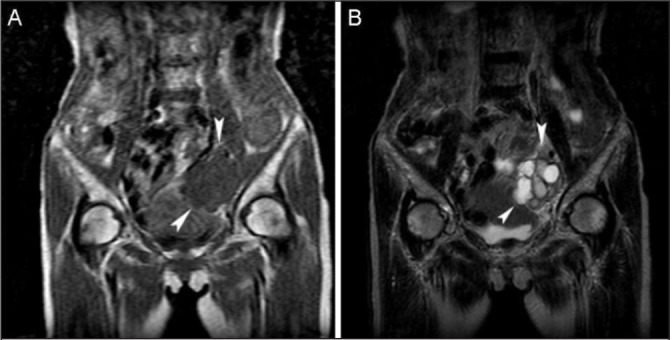
Coronal magnetic resonance imaging (MRI) of the pelvis at presentation: T1 weighted MRI showing an inhomogeneous low intensity mass (A) and T2 weighted MRI showing internal multicystic components with homogeneously high intensities (B)

Abdominal computed tomography (CT) and pelvic magnetic resonance imaging (MRI) revealed a mass approximately 5cm × 7cm × 7cm in size in the left pelvic wall (Figs 1 and 2). No nodal or distant metastases were detected using CT or MRI. Chest radiography was normal.

**Figure 3:**
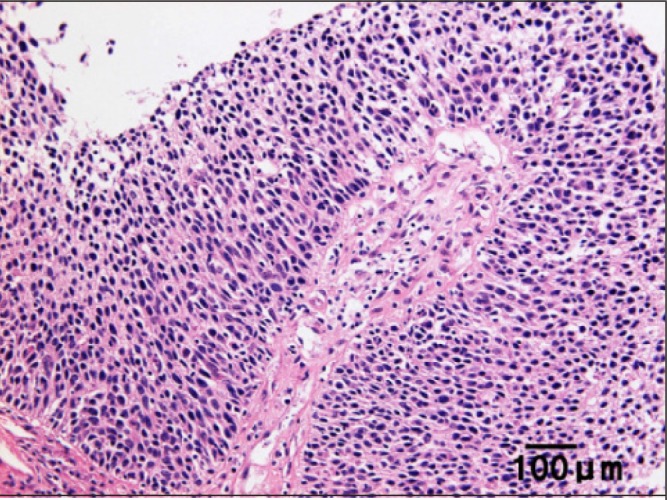
Photomicrograph (haematoxylin and eosin stain; 20× magnification) of the biopsied specimen from the tumour showing the characteristic cell pattern of transitional cell carcinoma

Laparotomy disclosed a retroperitoneal mass extending caudally to the lesser pelvis. A normal-appearing left ovary and left uterine tube were located inside the mass. The other intrapelvic organs were normal. A small amount of ascites was observed without intraperitoneal implants. The mass could not be dissected and therefore only incision biopsy was performed. Histology revealed a transitional cell carcinoma (TCC) without any ovarian tissues (Fig 3). Cytology of the ascites was negative. Postoperatively, cystoscopy and intravenous pyelography were performed with negative results. On the basis of these findings, a diagnosis was made of primary retroperitoneal TCC.

In November 2001 oral chemotherapy with tegafur-uracil (UFT) at a dose of 300mg/m^2^/day was initiated in an outpatient setting because the patient and her family hoped to avoid any invasive treatment. At two months from the initiation of chemotherapy, the patient complained of swelling in the left leg in spite of a slight decrease in serum CA125 level (304.5u/ml). One month later, abdominal CT revealed a growing mass 8cm × 8cm × 8cm in size but no nodal or distant metastases. Serum CA125 was again increasing (351.0u/ml). Radiotherapy using a three-dimensional conformal technique was initiated in March 2002.

At the end of radiation of 50Gy in 25 fractions, oedema in the left leg had almost disappeared and there was a prominent decrease in serum CA125 (25.5u/ml) but the tumour showed almost no change in size on CT. The chemotherapy was continued during and after the radiotherapy. From February 2003 the drug was administered at a smaller dose of 100mg/m^2^/day because of leucopoenia.

**Figure 4:**
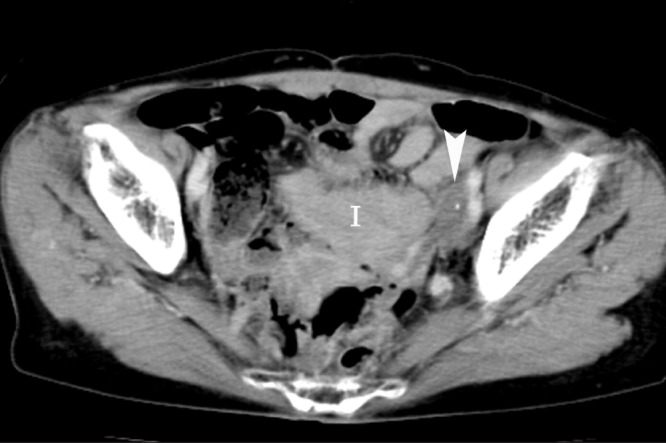
Post-contrast computed tomography performed 20 months after the initiation of radiotherapy showing a small area of low density with spotty calcifications (arrowhead) I = intestinal loops

In November 2003, at 20 months from initiation of radiotherapy, the tumour had almost disappeared except for a tiny nodule detected on follow-up CT (Fig 4). Until that time, serum CA125 had been within the normal range for 19 months. Considering the possibility of a residual tumour, the chemotherapy was continued.

In November 2006 the patient received a left mastectomy for breast cancer followed by a single course of adjuvant chemotherapy. In January 2007 she underwent adjuvant hormonal therapy.

In December 2008 pelvic CT revealed the tiny nodule still present without any change in appearance and size. Although tumour markers were not examined at that time, the tumour seemed to have already attained complete remission. Consequently, chemotherapy was ended. From the initiation of chemotherapy, interruptions had occurred four times owing to leucopoenia or adjuvant chemotherapy for breast cancer.

The last follow-up CT in May 2012, ten years from initiation of radiotherapy, revealed no recurrence. The patient had been well and was receiving adjuvant hormonal therapy.

## Discussion

Primary retroperitoneal TCCs are extremely rare entities. We could identify only three single case reports published in the last five decades.^1−3^ Two of these cases involved radiotherapy alone at a total dose of 50Gy in 25 fractions and radiotherapy at an unspecified dose, and the remaining case involved surgery and adjuvant chemotherapy. Despite this, all patients died of the tumour 8−24 months after diagnosis or treatment. In our case, however, the patient has remained well with no recurrence over the ten years since radiotherapy. An explanation for the difference in the effectiveness of radiotherapy between the previous case and ours is that in our case, the concurrently administered chemotherapeutic agent acted as a radiosensitiser.

Serum CA125 is a clinically useful marker of tumour response or recurrence but not very helpful in differentiating the exact origin of the tumour. In our case, serum CA125 was increased before radiotherapy but had decreased steeply at the end of radiotherapy. At that time, the tumour had not changed in size but was finally completely remitted. Therefore, in our case, the decline in CA125 seemed to be a predictor for tumour response.

The tumour had almost disappeared in November 2003. Furthermore, during the subsequent 8.5 years until the last follow-up appointment in May 2012, there was no recurrence detected on CT or by serum CA125 levels. Accordingly, complete remission of the tumour seemed to have been attained as late as November 2003.

Concerning the origins of this retroperitoneal tumour with cystic components, the following five major hypotheses were proposed by Pearl *et al*: (i) heterotopic ovarian tissue; (ii) monodermal variant of teratomas; (iii) embryonal urogenital remnants; (iv) intestinal duplication; and (v) coelomic metaplasia.^4^ According to Hansmann and Budd,^5^ the most provable origin for our case seems to be embryonal urogenital remnants. However, owing to the possibility of a sampling error in biopsy and the pathological findings in our case, the hypothesis of the tumour originating from heterotopic ovarian tissue or a monodermal variant of a teratoma could not be totally excluded.

## Conclusions

Because of the paucity of experience in the treatment of primary retroperitoneal TCCs, each patient should be individualised. Our patient was cured with radiotherapy and oral chemotherapy but the role played by continued administration of the oral drug following radiotherapy remains unknown. Nevertheless, based on the success of our case, radiotherapy with concurrent oral chemotherapy should be considered as an option for unresected cases.
